# Effects of Titanium Mesh Surfaces-Coated with Hydroxyapatite/β-Tricalcium Phosphate Nanotubes on Acetabular Bone Defects in Rabbits

**DOI:** 10.3390/ijms18071462

**Published:** 2017-07-07

**Authors:** Thuy-Duong Thi Nguyen, Tae-Sung Bae, Dae-hyeok Yang, Myung-sik Park, Sun-jung Yoon

**Affiliations:** 1 Department of Dental Biomaterials, Institute of Oral Bioscience and Institute of Biodegradable Material, School of Dentistry, Chonbuk National University, Jeonju 54896, Korea; drduongendo@gmail.com (T.-D.T.N.); bts@jbnu.ac.kr (T.-S.B.); 2 Institute of Cell and Tissue Engineering, Department of Biomedical Sciences, College of Medicine, The Catholic University of Korea, Seoul 06591, Korea; yang770606@gmail.com; 3 Department of Orthopedic Surgery, Research Institute of Clinical Medicine of Chonbuk National University, Biomedical Research Institute of Chonbuk National University Hospital, Jeonju 54907, Korea; mspark@jbnu.ac.kr

**Keywords:** titanium mesh, surface modification, hydroxyapatite, β-tricalcium phosphate, bone defect, acetabular defect, total hip arthroplasty, bone tissue engineering, orthopedic surgery

## Abstract

The management of severe acetabular bone defects in revision reconstructive orthopedic surgery is challenging. In this study, cyclic precalcification (CP) treatment was used on both nanotube-surface Ti-mesh and a bone graft substitute for the acetabular defect model, and its effects were assessed in vitro and in vivo. Nanotube-Ti mesh coated with hydroxyapatite/β-tricalcium phosphate (HA/β-TCP) was manufactured by an anodizing and a sintering method, respectively. An 8 mm diameter defect was created on each acetabulum of eight rabbits, then treated by grafting materials and covered by Ti meshes. At four and eight weeks, postoperatively, biopsies were performed for histomorphometric analyses. The newly-formed bone layers under cyclic precalcified anodized Ti (CP-AT) meshes were superior with regard to the mineralized area at both four and eight weeks, as compared with that under untreated Ti meshes. Active bone regeneration at 2–4 weeks was stronger than at 6–8 weeks, particularly with treated biphasic ceramic (*p* < 0.05). CP improved the bioactivity of Ti meshes and biphasic grafting materials. Moreover, the precalcified nanotubular Ti meshes could enhance early contact bone formation on the mesh and, therefore, may reduce the collapse of Ti meshes into the defect, increasing the sufficiency of acetabular reconstruction. Finally, cyclic precalcification did not affect bone regeneration by biphasic grafting materials in vivo.

## 1. Introduction

The management of severe acetabular bone defects in revision reconstructive orthopedic surgery is challenging. Many patients show failure of revisions or serious acetabular bone defects in total hip arthroplasty. In particular, severe bone loss with combined cavitary and segmental defects has been traditionally treated using structural allografts, highly-porous metal shells with or without cages, custom triangular cups, or trabecular metal augments [1,2,3].

Among the materials used for bone defect repair in adult reconstructive revision surgery, metallic mesh provides favorable biomechanical properties. Moreover, flexible reconstruction meshes provide a stable cavity for impaction bone grafting reconstruction and cup fixation [4]. Previous studies have shown that surface-modified titanium mesh can be applied for dental and orthopedic implants in the clinical setting.

Chemical modification of titanium (Ti) surfaces, such as coating with hydroxyapatite (HA), may be an effective method to enable activation of in vivo response to accelerate bone conduction [5,6,7]. In our previous studies, calcium phosphate was precoated on a nanotubular Ti substrate by a cyclic precalcification process [8]. This is a coating that has shown high bioactivity in simulated body fluid (SBF) by inducing the formation of HA. Thus, an HA layer mimicking the natural material was used to coat the Ti substrate, resulting in increased osteoblast responses, bone conduction, and bone integration [8,9,10]. Importantly, this cyclic precalcification treatment is expected to facilitate bone formation on nanotube-surface Ti-mesh.

In addition to the use of Ti-mesh to support bone defects, filling the void by suitable bone-graft substitutes is also necessary in reconstructive orthopedic surgery. In this study, a biphasic bioceramic of 6HA:4β-tricalcium phosphate (β-TCP) was manufactured by sintering method with a porous structure. In order to increase the bioactivity of this grafting material, cyclic precalcification was also applied by soaking the bioceramic in Ca-containing solution and P-containing solution sequentially.

Therefore, in this study, cyclic precalcification (HA/β-TCP) treatment was used on both nanotube-surface Ti-mesh and bone graft substitute (β-TCP) for the acetabular defect model, and its effects were assessed in vitro and in vivo.

## 2. Results

### 2.1. Effects of Cyclic Precalcifcation on the Bioactivity of Ti Mesh

**Figure 1:**
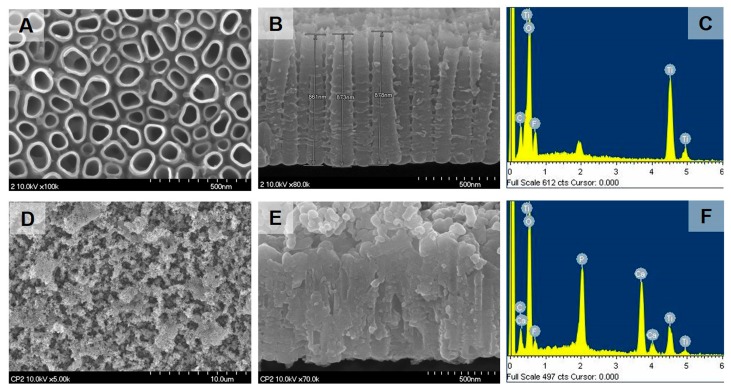
Field emission scanning electron microscopy (FE-SEM) and EDS analysis of the nanotube layer (**A**–**C**) and precalcified nanotube layer (**D**–**F**) on a Ti mesh.

Figure 1A,B shows the morphology of nanotubular arrays on Ti mesh used for applying the cyclic precalcification process. Each nanotube had an independent tubular structure and the diameter was increased from top to bottom with a hollow internal structure, and an approximate length of 870 nm. These nanotubes were then covered by a homogenous layer of calcium phosphate precipitates through a cyclic precalcification treatment (Figure 1D). These grain-like precipitates were interlocked with the porous nanotubular surfaces, and partially penetrated into the nanotubes (Figure 1E). Energy dispersive X-ray (EDS) analysis also confirmed the nanotubular Ti surface was were covered by a rich calcium phosphate layer (Figure 1F).

**Figure 2:**
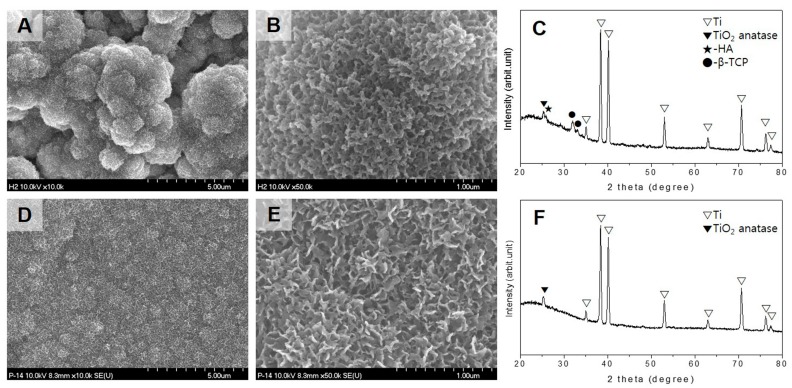
Bioactivity of Ti mesh after immersion in simulated body fluid (SBF) at 37 °C. Precalcified nanotubular Ti mesh (CP-AT) showed large and dense aggregates of crystalline structures on the surface (**A**,**B**) and hydroxyapatite (HA) and β-tricalcium phosphate (β-TCP) peaks on XRD analysis after three days of immersion (**C**). Nanotubular Ti mesh (AT, anodized Ti mesh) showed small aggregates of crystalline structures (**D**,**E**) after 14 days of immersion; none of the HA peaks were observed on XRD analysis (**F**).

The bioactivity of the Ti mesh was assayed through SBF-immersion testing at 37 °C. Titanium anodized surfaces (AT) are bioactive used in this test as the control group. Precalcified anodized Ti mesh (CP-AT) showed early bioactivity after three days of immersion (Figure 2A–C). In these figures, large spherical aggregates containing dense nanosized flake-like crystals were found throughout the entire Ti surface; these crystals were characterized as a mixture of HA and β-TCP crystals on X-ray diffraction (XRD) analysis. Additionally, on the nanotubular surface, crystalline structures were detected after 14 days of immersion. Figure 2D,E demonstrate the presence of a rather smooth and homogenous crystalline surfaces; however, no HA or β-TCP peaks were observed by XRD analysis due to the thin layer. Thus, precalcification treatment played a major role in increasing the bioactivity of the nanotubular surface.

**Figure 3:**
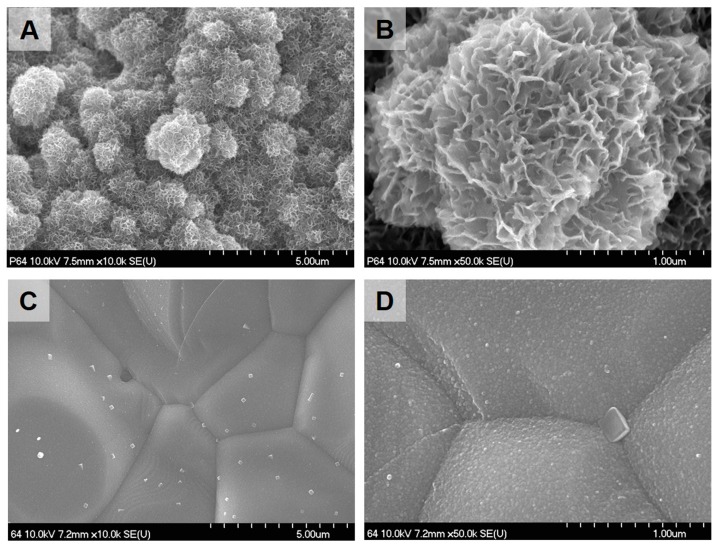
Bioactivity of biphasic ceramic immersed in SBF at 37 °C for three days. Precalcified biphasic ceramic (CP-BC) showed large, dense aggregates of crystalline structures on the surface (**A**,**B**); untreated biphasic ceramic (BC) did not show any changes on the surface (**C**,**D**).

The bioactivity of biphasic ceramic (BC) also was checked by SBF immersion tests (Figure 3). After three days of immersion, cyclic precalcified biphasic ceramic (CP-BC) samples were densely covered in HA protuberances over the entire surface; this phenomenon was not observed in untreated biphasic ceramic, suggesting that cyclic precalcification not only affected the Ti substrate but also the biphasic ceramic surface.

### 2.2. Effects of Cyclic Precalcification on the Flexural Properties of Ti Mesh

**Figure 4:**
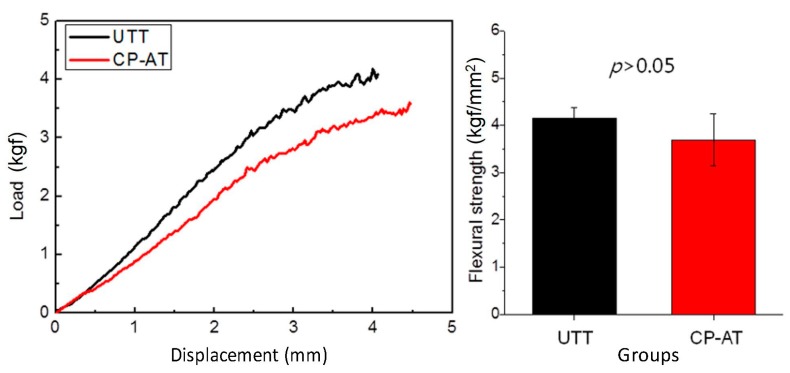
Three-point flexural testing of untreated Ti mesh (UTT) and cyclic precalcified Ti mesh (CP-AT).

To evaluate the effects of cyclic precalcification on the flexural strength of Ti mesh, three-point bending tests were performed for the samples of each Ti mesh group (*n* = 3). Figure 4 shows the load deflection curves and mean flexural strength of each group. The mean flexural strength values were 4.15 ± 0.24 kgf/mm^2^ in the untreated Ti mesh (UTT) group, and 3.69 ± 0.55 kgf/mm^2^ in the precalcified anodized Ti mesh (CP-AT) group. There was no significant difference between the two groups (*p* > 0.05).

### 2.3. Effects of Cyclic Precalcification on Bone Apposition on Ti Mesh

**Figure 5:**
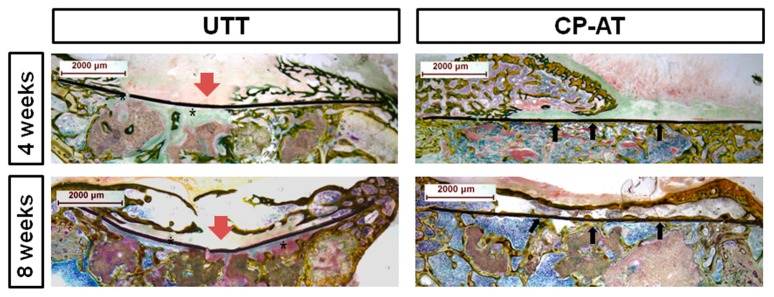
Bone healing pattern of the acetabular defects at four and eight weeks post-operation. Cross-section images (10×, Villanueva bone staining) through the center points of untreated Ti meshes and cyclic precalcified-Ti mesh. Red arrows: Ti mesh deformations; asterisks: soft tissue intervention under Ti mesh; black arrows: new bone in contact with Ti mesh.

To observe the bone apposition on Ti mesh, Ti mesh-defect biopsies were perpendicularly sectioned to the plane of the Ti mesh through the center point. Figure 5 represents the histological images of these sections of UTT and CP-AT groups at four and eight weeks post-operation. As shown in the image, the UTT group exhibited deformation of the Ti mesh at both time points (red arrows). The UTT mesh tended to collapse into the defect, which did not occur in the CP-AT group. Additionally, the UTT mesh seemed to be covered mostly by soft tissue intervention, and a thin layer of new bone formed separately underneath this soft tissue. Another new bone layer was observed in contact with CP-AT mesh, even in the center of the mesh. In both two groups, over-growth of bone formed on the Ti meshes. Bone grew on the UTT mesh as small pieces and was invaded by connective tissue. In the CP-AT group, a trabecular laminate covered partly on the mesh surface at four weeks, and completely at eight weeks.

**Figure 6:**
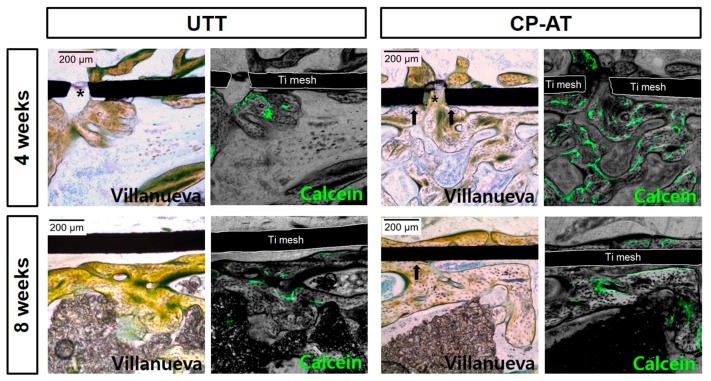
Representative images of bone tissue regeneration under untreated and treated Ti meshes at four and eight weeks by conventional histological staining (left, 100×, Villanueva bone staining, light microscopy). The newly-formed bone was in direct contact with the grafting materials in some positions (arrows). Mineralized bone (unstained) and seams of osteoids (yellowish green) are distinguished. Asterisks: hole of Ti meshes; black arrows: new bone in contact with Ti meshes. Calcein (green), which was injected two weeks before sacrifice, was also observed by confocal laser scanning microscopy in the same sections (right).

Figure 6 showed high-magnification images of bone regeneration under untreated and treated Ti meshes. The images confirmed that new bone formation was increased, and the new bone had better contact (arrows) with treated Ti meshes than with untreated meshes. The new bone nearly filled the holes of CP-AT meshes. On Villanueva-stained images, osteoids were stained green or jade green, whereas the mineralized bone matrix was unstained (white) or light green [11]. The newly-formed bone layers under CP-AT meshes were superior with regard to the mineralized area at both four and eight weeks, as compared with those under untreated Ti meshes. Moreover, to observe the de novo bone dynamics under Ti meshes, these specimens were studied by fluorochrome labeling, two weeks prior to sacrifice. In animals sacrificed at four weeks a stronger stained bone staining was observed in the CP-AT group compared with that in the UTT groups. However, these labeling patterns were no different in animals sacrificed at eight weeks, suggesting that the mode of ossification did not differ between groups at later healing times.

### 2.4. Effects of Cyclic Precalcification on the Bone Regeneration Ability of Biphasic Ceramic

**Figure 7:**
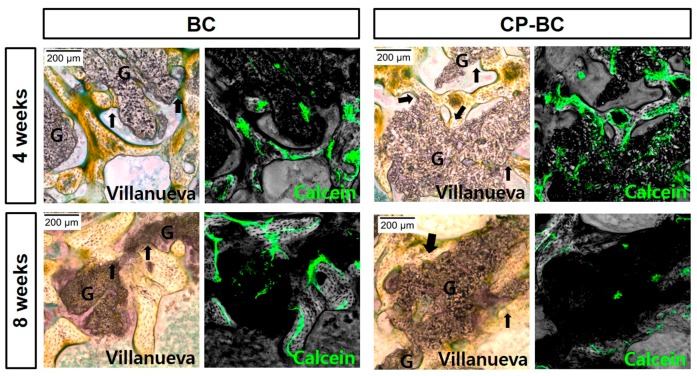
Representative images of bone tissue regeneration inside defects with untreated (BC) and treated (CP-BC) biphasic grafting materials at four and eight weeks by conventional histological staining (left, 100×, Villanueva bone staining, light microscopy). The newly-formed bone was in direct contact with the grafting materials in some positions (arrows). Mineralized bone (unstained) and seams of osteoids (yellowish green) are shown. G indicates grafting materials. Calcein (green), which was injected two weeks before sacrifice were also observed by confocal laser scanning microscopy in the same sections (right).

Figure 7 showed the bone formation pattern inside defects treated by biphasic grafting materials (6:4 HA:βTCP). As shown by the Villanueva staining images, all groups exhibited the same ossification mode, with newly-formed bone between grafting materials, partly integrated with materials.

**Figure 8:**
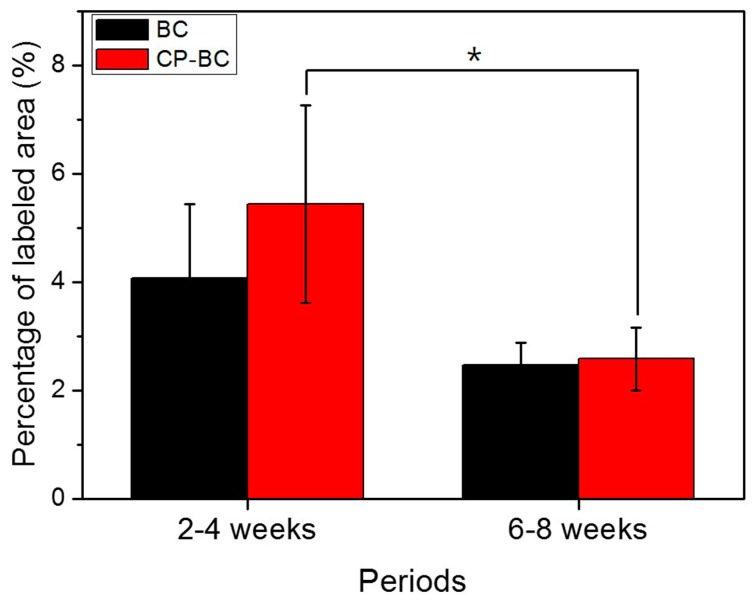
Percentage of mineralized area labeled by calcein green in relation to the determined area at different periods in defects exposed to untreated (BC) or treated (CP-BC) biphasic grafting materials. * Significantly different between groups (*p* < 0.05).

Calcein staining at two weeks prior to sacrifice showed the mineralization inside the grafting materials. Based on these calcein labels, we determined the percent mineralized area in relation to the determined area for different period: 2–4 and 6–8 weeks (Figure 8). Active bone regeneration at 2–4 weeks was stronger than at 6–8 weeks, particularly with treated biphasic ceramic (*p* < 0.05). However, the differences between the two groups was not significant (*p* > 0.05).

At eight weeks, both groups showed the formation of a bony bridge from below the cortical bone layer to the grafting materials (Figure S1). Calcein labeling and Villanueva staining illustrated that bone formation occurred outward; the first formed bone was replaced by mature bone and new osteoids developed on this bone.

## 3. Discussion

The reconstruction of large acetabular bone defects is a challenge in revision total hip arthroplasty [12]. Flexible reconstruction meshes can be used to convert uncontained defects into contained defects, thus providing a stable cavity for impaction bone grafting reconstruction and cemented cup fixation. Alternatives include impaction allografting with cement with containment created by the use of rim meshes, the use of bulk allografts, bilobed acetabular components, jumbo acetabular components, high placement of the hip center of rotation, or a reconstruction cage [13,14].

This present study was undertaken to assess the effects of cyclic precalcification treatment on titanium mesh and grafting material for acetabular reconstruction in vitro and in vivo. This study is the first to investigate surface-modified titanium mesh of acetabular defects in an animal model.

Cyclic precalcification is the repeated process of loading Ca and P on materials in order to improve the substrate’s bioactivity, providing a better environment for osteoblastic cell attachment, proliferation, and differentiation in vitro, as well as bone regeneration in vivo [9,15]. Since this treatment is based on the precipitation of Ca and P, porous surfaces would be suitable to be used as a substrate to improve the loading of these elements. Nanotubular Ti mesh surfaces and porous biphasic grafting materials possess many empty spaces, which could become potential bioactive material carriers.

From SBF immersion tests, we concluded that precalcification increased the bioactivity of treated Ti meshes as previously reported [8,15]. Moreover, the application of cyclic precalcification on biphasic grafting materials also increased HA precipitation on treated materials during early healing. This phenomenon can be explained by the observation that local Ca–P supersaturation of the cyclic precalcified surface stimulates Ca–P nucleation and apatite-crystallization from SBF [8]. Thus, based on these investigations, cyclic precalcification improves the bioactivities of Ti meshes and biphasic grafting materials, implying that such modified materials may be suitable for acetabular reconstruction.

The titanium meshes used in this study were designed as perforated round plates with two wings for miniscrew fixation. This design keeps the specimen stable during healing in animal experiments and may allow diffusion of extracellular nutrients for bone regeneration [16]. However, the perforated meshes with multiple pores may permit soft tissue penetration and weaken the stability of the Ti meshes, resulting in membrane collapse [12,16]. In this current study, the untreated Ti mesh at both 4 and 8 weeks were found to be localized within the defects. However, the cyclic precalcification-treated nanotubular surface showed no deformation. Thus, we performed three-point flexural testing of Ti meshes with untreated and treated surfaces. The results clarified that this surface treatment did not affect the mechanical strength of the Ti meshes. Thus, the cause of deformation of the untreated Ti mesh was a lack of bony support underneath the mesh. During healing, UTT meshes were covered mostly by soft tissue intervention. A thin bone layer also was formed under the mesh, but was not in close contact with the mesh. Additionally, new bone layers grew along the TI meshes in the CP-AT groups with good bonding, supporting the Ti meshes from the pressure and invasion of the soft tissues. Moreover, a continuous bone layer also was found above treated meshes at eight weeks, separating the muscular layer from the defect and protecting the Ti meshes from collapsing. Therefore, the shape and structure of acetabular bone could be fully reconstructed during healing.

In order to evaluate bone regeneration under Ti meshes, calcein labeling was performed two weeks prior to sacrifice. This fluorochrome label binds with calcium, which remains available in the osteoid matrix and is then incorporated at sites of mineralization with HA crystals [17]. The label area shows mineralization during healing [17]. Thus, we estimated the mineralization area by labeling with calcein. Our findings showed that, the ossification activity was strong during the first four weeks of healing and slowed down during later stages of healing. This process was not affected by treatment of grafting materials. Although the treated biphasic ceramic showed higher bioactivity in only three days in vitro compared with untreated samples, in vivo bone regeneration on treated surfaces was not induced after four and eight weeks of healing*.* This result was consistent with the fact that the cyclic precalcification process improves the bioactivity of bioceramics during the early treatment phase.

Biphasic calcium phosphate (BC), a mixture of either β-TCP + HA or α-TCP + HA, is produced by balancing the more stable phase of HA and the more soluble molecule TCP [18]. In this current study, the biphasic bioceramic of 6HA:4β-TCP was used. During cyclic precalcification treatment, a layer of Ca–P containing both amorphous and crystalline phases (octacalcium phosphate—OCP) was coated onto this bioceramic [8]. The OCP crystal is a required precursor to biological apatite crystals [8,19]. Moreover, in a comparative study, β-TCP and HA bioceramics showed poor apatite precipitation both in vitro and in vivo. OCP formation ubiquitously occurred on all types of bioceramic surfaces, except on β-TCP [20]. Notably, this precipitation of calcium orthophosphates on bioceramic surface is more difficult in vivo than in vitro [20]. In vivo continuous solubilization of the precalcificated surface occurs during implanting until equilibrium between the physiological solutions and the modified surface of HA has been reached [18]. Intrinsic osteoinduction by calcium orthophosphate bioceramics is a result of adsorption of osteoinductive substances on their surface [21]. Thus, the bioactive surface may not be as effective during later healing stage, as supported by the similar ossification patterns of untreated and treated biphasic ceramics at four and eight weeks. Additional studies with larger sample sizes and shorter experimental periods are needed to clarify this hypothesis.

In this study, the critical size defects of 8 mm were used to investigate the bone regeneration treated by the Ti meshes and grafting materials. This is the smallest-sized tissue defect that will not heal spontaneously [22]. In the clinical setting the bone defects might be much larger than this size, thus, further studies with larger sample sizes and larger models should be conducted to translate these preliminary results into clinical applications.

## 4. Materials and Methods

### 4.1. Titanium Mesh Preparation 

**Figure 9:**
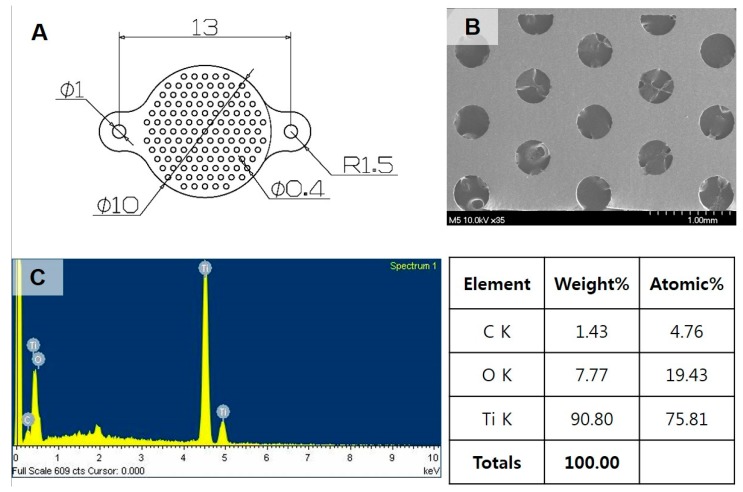
Characterization of pristine Ti mesh. (**A**) Technical drawing; (**B**) Field emission scanning electron microscopy (FE-SEM) images: perforated Ti mesh with multiple holes; (**C**) EDS analysis of the chemical composition of the Ti mesh.

Experimental Ti-mesh consisted of custom-made, round plates (10 mm in diameter) with two wings in two sides for miniscrew fixation (Figure 9A). The mesh were cut from Neo Titanium mesh (Neobiotech Co., Ltd., Los Angeles, CA, USA) with a thickness of 90 μm. Ti mesh was perforated with multiple holes (40 μm in diameter; Figure 1B). Prior to cyclic precalcification, a fresh oxide layer was obtained by acid-etching with a mixture (HNO_3_:HF:H_2_O = 12:7:81) for 10 s, and a nanotube layer was then prepared on the Ti substrate by an anodizing process in glycerol solution at 20 V for 1 h [8].

### 4.2. Porous Biphasic (6HA:4β-TCP) Bioceramic Manufacturing

A mixture of HA (Sigma-Aldrich, St. Louis, MO, USA) and β-TCP (Sigma-Aldrich) with an additional amount of silica (SiO_2_; 1 wt %) was prepared to yield a biphasic ceramic with an HA/β-TCP ratio of 6:4 by the sintering method. Silica dioxide was used to form the interfacial bonding between components because silica tends to form low melting compounds at the sintering temperatures used in this study (1300 °C). To make a biphasic ceramic with a 6:4 ratio, 6:4 powder containing 30 wt % carbon powder (Indocarb Corporation Inc., Pittsburgh, PA, USA) was die-pressed in a cylindrical mold (diameter: 15 mm) under a pressure of 1 MPa at ambient temperature. The obtained pellets were calcinated at 600 °C for 6 h, following by sintering in air at 1300 °C for 4.5 h. After sintering, the ceramic pellets were ball milled and sieved through a 400 µm metal sieve mesh.

### 4.3. Cyclic Precalcification 

As has been described in previous studies [8,9], cyclic precalcification of the anodized Ti mesh and the biphasic bioceramic was conducted in soaking the specimens in turn for 1 min with 0.05 M NaH_2_PO_4_ solution at 80 °C and saturated Ca(OH)_2_ solution at 100 °C. This process was repeated for 30 cycles. Finally, to stabilize the formed structure and remove any remaining impurities, the cyclic precalcification-treated samples were heated at 500 °C for 2 h.

### 4.4. Surface Characterization

The morphology and chemical composition of the sample surface was observed by field emission scanning electron microscopy (FE-SEM, S-4700, Hitachi, Tokyo, Japan) equipped with EDS. The phase composition was analyzed by XRD (Multi-Purpose High-Performance X-ray Diffractometer, X’pert Power, PANalytical Co., Tokyo, Japan), with a scanning rate of 4°/min in the range of 20–80°.

### 4.5. Bioactivity Test

The in vitro bioactivity of the cyclic precalcification-treated Ti mesh and biphasic ceramic was evaluated by immersing the specimens in SBF at 37.5 °C and daily checking the apatite crystal formation by FE-SEM up to 14 days. SBF with ionic concentrations similar to human blood plasma were prepared by mixing 0.185 g/L CaCl_2_·2H_2_O, 0.09767 g/L MgSO_4_, 0.35 g/L NaHCO_3_, and Hank’s balanced solution (H2387; Sigma Chemical Co., Saint Louis, MO, USA) and buffering the solution at pH 7.4. HA formation on the surface of specimens was investigated by XRD.

### 4.6. Three-Point Flexural Testing on Ti Mesh

Three-point bending tests were conducted using an INSTRON universal testing machine (Instron 4201, Instron Corporation, Norwood, MA, USA) at a crosshead speed of 2 mm/min. The Ti meshes with untreated and treated surfaces were prepared with a thickness of 90 μm and then cut to a size of 23 mm × 3 mm. Six samples for each group were tested at room temperature and the load deflection curve of each sample was recorded. The flexural strength σ (kgf/mm^2^) was calculated by using the following equation:

(1) $$\sigma =\frac{3pL}{2bh^{2}}$$

where *p* is the load in kgf, *L* is the distance between supports (20 mm), *b* is the width (3.0 ± 0.1 mm) and *h* is thickness (90 ± 5 μm).

### 4.7. Animals

Prior to in vivo tests, all specimens were sterilized using ethylene oxide gas. To evaluate the effects of cyclic precalcification on Ti mesh and biphasic ceramic materials, four in vivo experimental groups were set up (Table 1). In groups 1 and 2, to assess the effects of the cyclic precalcification process on Ti meshes, cyclic precalcified Ti anodized Ti mesh (CP-AT) and untreated Ti mesh (UTT) were used to treat the defect with a commercial porous bone substitute product (U-bone, granule type; Biorigin, Korea). The grafting material consisted of the β-TCP granule (size: 1.0 mm × 1.5 mm). In groups 3 and 4, in order to compare between untreated (BC) and treated biphasic ceramics (CP-BC), treated Ti-meshes (CP-AT) was used.

Eight adult male New Zealand White rabbits (12–13 weeks of age, weighing ~3 kg) were used (two control, with 16 acetabular defects and two defects/group/period). One rabbit randomly received two different treatment groups on each acetabular defect. This study was conducted in compliance with the principles of the Declaration of Helsinki. The study protocol was approved by the Institutional Animal Care and Use Committee of the Chonbuk National University Laboratory Animal Center, Jeonju, Korea (Approved Number: CBU 2014-00050, 1 July 2014).

### 4.8. Operation Process

Prior to bilateral acetabular defect surgery, general anesthesia was induced by injection of ketamine hydrochloride (ketamine; Yuhan Corporation, Seoul, Korea; 35 mg/kg) and xylazine hydrochloride (Rompun, Bayer, Korea; 5 mg/kg) via an ear vein. Hindquarters were shaved, draped, and disinfected with betadine scrubs. Additional local anesthesia was given at the surgical site using 1% lidocaine with epinephrine (1:100,000) to reduce bleeding under the skin. Bilateral acetabular defects were made to mimic bone defects of revision total hip arthroplasty, as previously described [23]. A skin and fascia incision was made over the iliac crest, and the dissection was carried out along the ilium toward the hip joint, exposing the superior dome of the acetabulum to create defect. An 8 mm diameter defect through the cortical layer and trabecular bone layer was then created on this area using a trephine bur connected to an endodontic motor (X-SMART; Densply, Tokyo, Japan) under irrigation with sterile saline. The opposite cortical layer was carefully preserved to prevent perforation into the pelvic cavity. The periphery of the defect was assessed and washed to remove the remaining bone fragments or bone chips. The defects were completely filled with grafting materials. The meshes were placed to completely cover the bone defects, extending onto the surrounding bone margins at least 1 mm. Then, two screws were used to secure the mesh through its wings. The iliac muscle was replaced and sutured with bioabsorbable silk (4-0 Polyglactin 910 [Vicryl], Ethicon, Livingston, UK.). Finally, a 4/0 suture silk (4/0 Black silk, Ailee Co., Busan, Korea) was used to approximate the skin. In order to prevent infection and control pain, antibiotics (Amikacin; Samu Median, Yesan, Korea) (0.15 mL/kg) and analgesics (Nobin, Bayer, Seoul, Korea) were administrated intramuscularly for three days at 0, 24, and 48 h, postoperatively.

To clarify the bone formation activity inside the defects, all animals were injected intraperitoneally with calcein (Sigma-Aldrich) at 1.25 mg/kg body weight two weeks prior to sacrifice.

The animals were sacrificed at four or eight weeks by an overdose of thiopental (thiopental sodium; ChoongWae Pharma, Seoul, Korea). The hemipelvis with the experimental defects and materials were dissected, cut to 2 × 2 cm blocks, and fixed with 10% neutral buffered formalin.

### 4.9. Histological and Fluorescent Analysis

The specimens were stained with Villanueva solution (Polysciences, Inc., Hirschberg, Germany), dehydrated in a series of increasing concentrations of ethanol (80%, 90%, 95%, and 100%), and embedded in methyl methacrylate (MMA monomer, Yaruki Pure Chemicals Co., Ltd., Kyoto, Japan). Resin blocks containing samples were perpendicularly sectioned to the plane of the Ti membrane through the center point using a low-speed diamond cutter to a thickness of 0.5 mm. Finally, the specimens were ground to 40 µm thickness to prepare for hard tissue examination. The prepared specimens were examined using low-magnification optical microscopy (EZ4D, Leica Microsystem, Wetzlar, Germany) at 10×, and 30×, and a high-magnification optical microscopy (DM2500, Leica Microsystem) at 100×.

The calcein green labels on these histological sections also were visualized using a confocal laser scanning microscope (CLSM 510 Meta, Zeiss, München, Germany) at a magnification of 100× with excitation of 488 nm and emission at over 560 nm.

Fluorochrome staining was initiated to detect new bone formation and mineralization two weeks prior to sacrifice. Three random images obtained inside the defect sites by CLSM at 100× magnification (513 × 513 pixels). The percentage of pixels labeled with calcein green was evaluated on each image using an image-analysis computer program (ImageJ 1.46; National Institutes of Health, Bethesda, MD, USA). The mean value of the six measurements was calculated per group (three measurements/defect, two defects/group) [24].

### 4.10. Statistic Analysis

The flexural strength (kgf/mm^2^) and the relative mineralized area (%) data were expressed as means ± standard deviations. Statistical analysis was performed using one way ANOVA with Tukey’s post-hoc test in SPSS software (version 12.0; SPSS, Chicago, IL, USA). Differences with *p* values of less than 0.05 were considered as statistically significant.

## 5. Conclusions

Despite the limitations of this study, we concluded that cyclic precalcification improved the bioactivity of Ti meshes and biphasic grafting materials. Moreover, the precalcified nanotubular Ti meshes could enhance early contact bone formation on the mesh and, therefore, may reduce the collapse of Ti meshes into the defect, increasing the sufficiency of acetabular reconstruction. Finally, cyclic precalcification did not affect bone regeneration by biphasic grafting materials in vivo.

## Figures and Tables

**Table 1:** Groups for in vivo experiments.

Groups	Mesh Types	Grafting Materials
1	UTT: Untreated Ti mesh	Commercial β-TCP (U bone)
2	CP-AT: cyclic precalcified anodized Ti mesh	Commercial β-TCP (U bone)
3	CP-AT: cyclic precalcified anodized Ti mesh	BC: biphasic ceramic
4	CP-AT: cyclic precalcified anodized Ti mesh	CP-BC: cyclic precalcified biphasic ceramic

## Supplementary Materials

Supplementary materials can be found at www.mdpi.com/1422-0067/18/7/1462/s1.

## Abbreviations

Ti	Titanium
HA	Hydroxyapaptite
SBF	Simulated body fluid
β-TCP	β-Tricalcium phosphate
AT	Anodized Ti mesh
CP-AT	Cyclic precalcified anodized Ti mesh
XRD	X-ray diffraction
BC	Biphasic ceramic
CP-BC	Cyclic precalcified biphasic ceramic
OCP	Octacalcium-phosphate
FE-SEM	Field emission scanning electron microscopy
EDS	Energy dispersive X-ray analysis
UTT	Untreated Ti mesh
CLSM	Confocal laser scanning microscopy
