# Supplementary material for: Effects of Titanium Mesh Surfaces-Coated with Hydroxyapatite/β-Tricalcium Phosphate Nanotubes on Acetabular Bone Defects in Rabbits
Source: Int J Mol Sci. 2017 Jul 7;18(7):1462. doi: 10.3390/ijms18071462 (PMC5535953; doi:10.3390/ijms18071462)
Supplement: Supplementary file 1 [file ijms-18-01462-s001.zip › ijms-202476-supplementary.pdf]

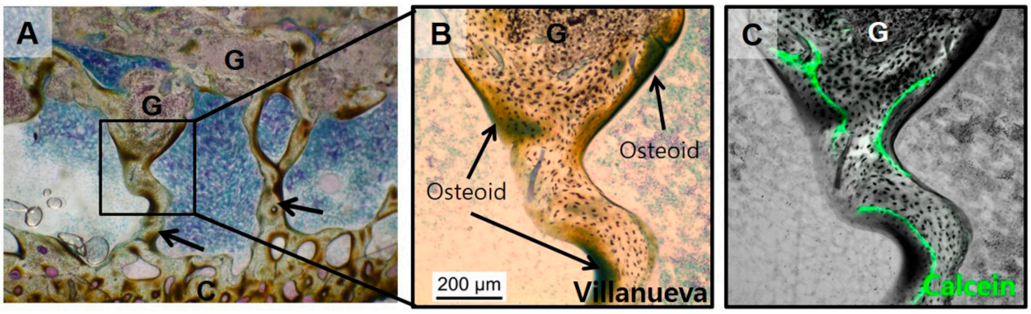

Figure S1. (**A**) The formation of bone bridges from below the cortical bone layer to the grafting materials at eight weeks post-operation (30×, Villanueva bone staining). (**B**) High magnification of the rectangle shown in (**A**) (100×). (**C**) Calcein labeling at the same position as that in (**B**) (100×). Arrows: bone bridges, G: grafting materials, C: opposite cortical bone layer.
